# WormCNN-Assisted Establishment and Analysis of Glycation Stress Models in *C. elegans*: Insights into Disease and Healthy Aging

**DOI:** 10.3390/ijms25179675

**Published:** 2024-09-06

**Authors:** Yan Pan, Zhihang Huang, Hongxia Cai, Zhiru Li, Jingyuan Zhu, Dan Wu, Wentao Xu, Hexiang Qiu, Nan Zhang, Guojun Li, Shan Gao, Bo Xian

**Affiliations:** 1 Laboratory of Aging Research, School of Medicine, University of Electronic Science and Technology of China, Chengdu 611731, China; yanpan@zohomail.com (Y.P.); wdwudan2024@163.com (D.W.);; 2 Institute for Toxicology, Beijing Center for Disease Prevention and Control, Beijing 100013, China

**Keywords:** Convolutional Neural Networks, computer vision, Glycation Stress, *C. elegans*, aging

## Abstract

Glycation Stress (GS), induced by advanced glycation end-products (AGEs), significantly impacts aging processes. This study introduces a new model of GS of *Caenorhabditis elegans* by feeding them *Escherichia coli* OP50 cultured in a glucose-enriched medium, which better simulates human dietary glycation compared to previous single protein–glucose cross-linking methods. Utilizing WormCNN, a deep learning model, we assessed the health status and calculated the Healthy Aging Index (HAI) of worms with or without GS. Our results demonstrated accelerated aging in the GS group, evidenced by increased autofluorescence and altered gene expression of key aging regulators, *daf-2* and *daf-16*. Additionally, we observed elevated pharyngeal pumping rates in AGEs-fed worms, suggesting an addictive response similar to human dietary patterns. This study highlights the profound effects of GS on worm aging and underscores the critical role of computer vision in accurately assessing health status and aiding in the establishment of disease models. The findings provide insights into glycation-induced aging and offer a comprehensive approach to studying the effects of dietary glycation on aging processes.

## 1. Introduction

In anti-aging medicine, Glycation Stress (GS) is increasingly recognized as a crucial factor influencing the human aging process [1,2]. As the primary source of energy, carbohydrates inevitably undergo glycation reactions with proteins during metabolism, leading to the formation of advanced glycation end-products (AGEs) [3]. This accumulation is especially evident in skin collagen and becomes increasingly pronounced with age, particularly in diabetics, where it manifests as decreased skin elasticity and visible signs of aging [4,5,6].

The worm *Caenorhabditis elegans* (*C. elegans*), widely used as a model organism, plays a crucial role in the study of various human diseases and aging processes [7]. Its short lifespan, transparent body, simple anatomy, and ease of genetic manipulation make it an ideal candidate for rapid, high-throughput disease modeling and drug screening [8,9,10]. This organism shares many essential biological pathways with humans, making it a valuable tool for understanding the molecular mechanisms of diseases and testing therapeutic interventions before they are applied to more complex models [11,12]. 

Specifically, glycation stress has significant implications for human populations, particularly those affected by conditions such as diabetes, metabolic syndrome, and age-related diseases. These individuals often experience common symptoms such as hyperglycemia, insulin resistance, and chronic inflammation, leading to complications like cardiovascular disease, neuropathy, and renal dysfunction [13]. Researchers have utilized *C. elegans* to explore the pathologies of neurodegenerative diseases [11] and metabolic disorders [9,14], illustrating its versatility and effectiveness in mimicking human disease states and evaluating potential treatments.

In addition to its application in modeling diseases, *C. elegans* can also be employed to assess healthy aging. Here, we introduce the concept of the Healthy Aging Index (HAI), which quantifies the biological health of aging in *C. elegans*. By comparing the predicted biological age of worms against normative data derived from standard lifespan conditions, the HAI provides a quantifiable measure of “healthy” aging. 

Expanding on the concept of GS, it not only includes the immediate stress reactions triggered by reducing sugars and aldehydes but also encompasses a cascade of non-enzymatic reactions that culminate in the production of AGEs [15]. These biochemical processes contribute to protein dysfunction, denaturation, and the decline of tissue protein functionality, ultimately inducing a variety of age-related degenerative changes [16].

However, despite its significance, there is currently a lack of a comprehensive worm model to study GS. Previous studies primarily focused on feeding worms AGEs derived from simple glucose–protein reactions, which fails to capture the multifaceted nature of glycation effects prevalent in human diets [17,18]. Establishing a comprehensive model of GS is crucial, as it allows for a deeper investigation into the molecular and physiological pathways altered by AGEs, providing insights that are vital for developing interventions to counteract aging and related diseases. This gap highlights the critical need for more representative models that can accurately reflect the variety and complexity of GS encountered in everyday human consumption [2,16,19,20].

Traditional approaches to studying GS in *C. elegans* often rely on biochemical assays and visual inspection, which, while informative, are limited in their ability to detect subtle, early-stage changes in organism physiology and behavior. These methods can overlook nuanced alterations preceding visible symptoms of aging or disease, potentially underestimating the impact of GS.

The integration of computer vision, particularly Convolutional Neural Networks (CNNs), into the study of aging and GS enriches our insights significantly. These technologies enable precise, automated worm morphology and behavior analyses, capturing subtle changes that traditional methods might miss [21,22,23]. Beyond simply observing, CNNs are crucial in biomedical applications such as drug discovery, where they help identify compounds that influence lifespan and health in worms [24,25,26]. They also aid in mapping neural activities and other dynamic biological processes, making them indispensable tools in modern aging research and related studies [27,28].

In summary, in response to the existing limitations, this work has developed a glycation-stressed *Escherichia coli* (*E. coli*) OP50 as a new method for establishing GS models in *C. elegans*. Using WormCNN to process reshaped images of *C. elegans*, we effectively assess premature aging and predict biological lifespan. This approach not only mirrors the chronic GS found in human diets more closely but also facilitates a deeper understanding of the mechanistic pathways influenced by dietary glycation. Our findings confirm that Glycation Stress (GS) precipitates premature aging, thereby establishing a model of GS in *C. elegans* that more accurately reflects the complexities associated with human glycation exposure validated by wet lab experiments.

## 2. Results

### 2.1. Lifespan Dataset Collection and Image Reshaping of C. elegans in a 384-Well Plate

To support the training of deep learning models for aging assessments, we monitored the lifespan of individual worms by placing each animal in a separate well of a 384-well plate. Our preprocessing workflow commenced with median filter denoising, which effectively minimized noise while retaining essential edge details of the worms. Subsequent to noise reduction, we applied histogram equalization to enhance the contrast of the images. Finally, the images were converted to 8-bit grayscale to streamline the processing and analysis stages.

**Figure 1:**
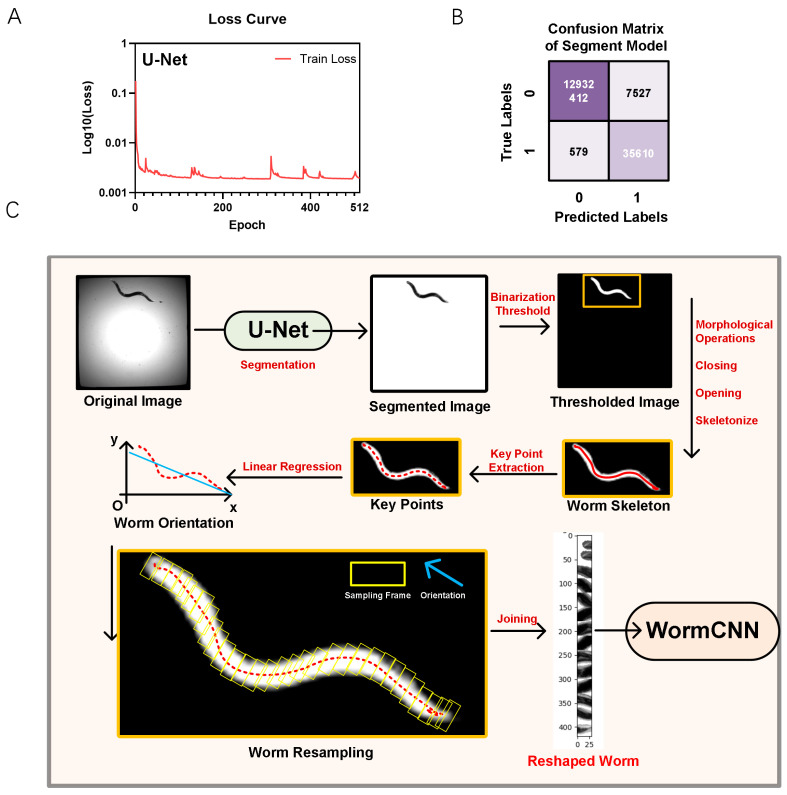
U-Net Model Performance and Workflow for Worm Segmentation and Reshaping. (**A**) The loss curve of the U-Net model during training showed a decrease in loss over 512 epochs. (**B**) Confusion matrix of the U-Net model. (**C**) Workflow diagram illustrating the process from original image acquisition to worm reshaping using U-Net segmentation, key points extraction, linear regression for orientation, and final resampling for analysis in WormCNN.

For segmentation, we employed a U-Net architecture. The U-Net model was trained on a dataset of pre-annotated images of worms in liquid culture media. As illustrated in Figure 1A, the final model’s loss plateaued after approximately 50 epochs, as depicted in the loss curve.

Once the worms were segmented, we focused on extracting their skeletons. This involved identifying key points along the length of each worm and using linear regression to determine their global orientation. 

The confusion matrix for the U-Net model’s performance on the test dataset is shown in Figure 1B. The model achieved a true positive rate (sensitivity) of 98.40% and a true negative rate (specificity) of 99.94%, demonstrating high accuracy in distinguishing between segmented and non-segmented areas of the images. The model’s overall accuracy was calculated to be 99.94%, with a precision (positive predictive value) of 82.55% and an F1-score of 89.78%. The detailed confusion matrix values are shown in Table 1.

By mapping the skeleton, we could quantify minor changes in shape and movement, providing valuable insights into the effects of GS and other factors being studied.

Figure 1C outlines the workflow from the original image acquisition to worm reshaping. It includes steps such as U-Net segmentation, key points extraction, linear regression for orientation, and final resampling for analysis in WormCNN.

Following the extraction of key points and orientation determination, the images were resampled and reshaped. This involved rotating and aligning the worm images based on the calculated orientation. The final reshaped images, standardized for further analysis, were then fed into the WormCNN for additional studies, such as aging and stress response assessments.

### 2.2. Elderly Classification and Regression Model with WormCNN

**Figure 2:**
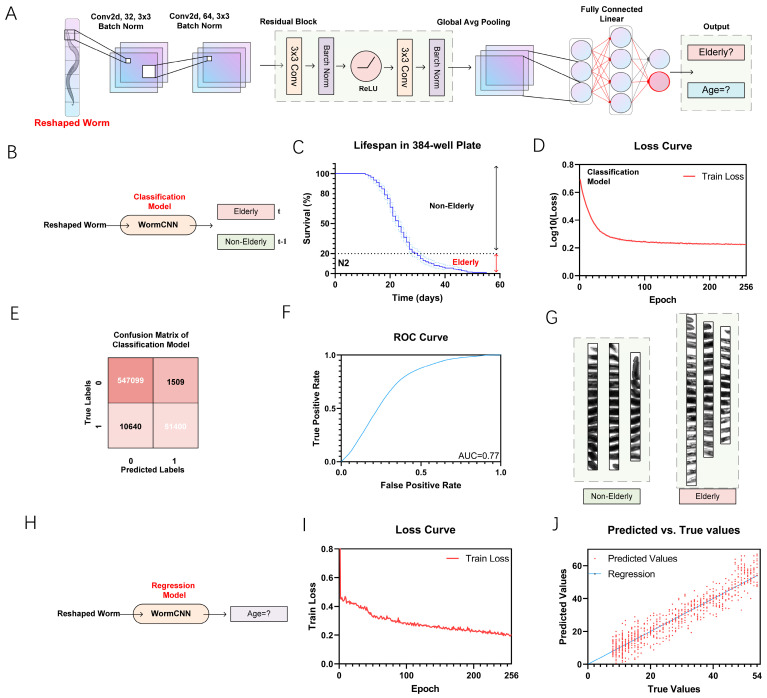
Comprehensive Overview of WormCNN’s Application in Classifying and Predicting the Lifespan of *C. elegans*. (**A**) Schematic of the WormCNN architecture showing various layers and their configurations used for processing the worm images. (**B**) Diagram illustrating the classification model workflow, where reshaped worm images are input into WormCNN to classify worms into elderly and non-elderly categories. (**C**) Lifespan curve in a 384-well plate showing the survival percentage of worms over time used to define the elderly threshold. The original image data can be found in Supplementary Table S1. (**D**) The loss curve for the classification model indicates the log-loss reduction as training progresses over epochs. (**E**) The confusion matrix of the classification model displaying the numbers of true positives, false positives, true negatives, and false negatives. (**F**) ROC curve illustrating the true positive rate against the false positive rate. (**G**) Reshaped worm images used in training the WormCNN, with images on the left representing non-elderly worms and those on the right depicting elderly ones. (**H**) Diagram illustrating the regression model workflow, where WormCNN estimates the actual ages of worms. (**I**) The loss curve for the regression model showing the decline in training loss, reflecting learning efficiency and model convergence. (**J**) A scatter plot compares predicted versus true age values, with a regression line indicating the accuracy of the age estimations. In this article, the predicted values are used as the “Healthy Aging Index” (HAI), which quantifies the deviation of each worm’s predicted age from its actual age.

We utilized WormCNN to develop a binary classification model for distinguishing between elderly and non-elderly *C. elegans* based on their lifespan when cultured in a 384-well plate (Figure 2A). The categorization was established using a 20% survival threshold, as depicted in the survival curve (Figure 2B,C). This curve illustrates the percentage of survival over time, with the 20% survival rate marked as the boundary separating the elderly from the non-elderly worms, and the loss curve was shown in Figure 2D.

WormCNN demonstrated exceptional performance in this classification task, achieving high precision and recall values (Figure 2E). 

The confusion matrix in Figure 2B highlights the model’s accuracy, showing a true positive rate of approximately 83.6% and a precision of about 97.3%, contributing to an overall accuracy of roughly 98.0%. The model also maintained a low false-positive rate, minimizing the misclassification of non-elderly elderly worms, as shown in Table 2.

The model’s discriminative power was further validated by the ROC curve, which exhibited an AUC of 0.77 (Figure 2F). Additionally, we adapted the model to perform regression analysis to estimate the actual age of the worms (Figure 2G,H). The training loss curve (Figure 2I) shows a steady decrease in loss, reflecting the model’s increasing accuracy in age prediction over the training epochs. This trend underscores the model’s effectiveness in learning age-related features from the worm images.

Moreover, the scatter plot (Figure 2J) shows a correlation between the predicted and true age values, with the regression line closely fitting the data points. This high correlation is quantified by a correlation coefficient of 0.804.

### 2.3. GS Modeling in C. elegans

**Figure 3:**
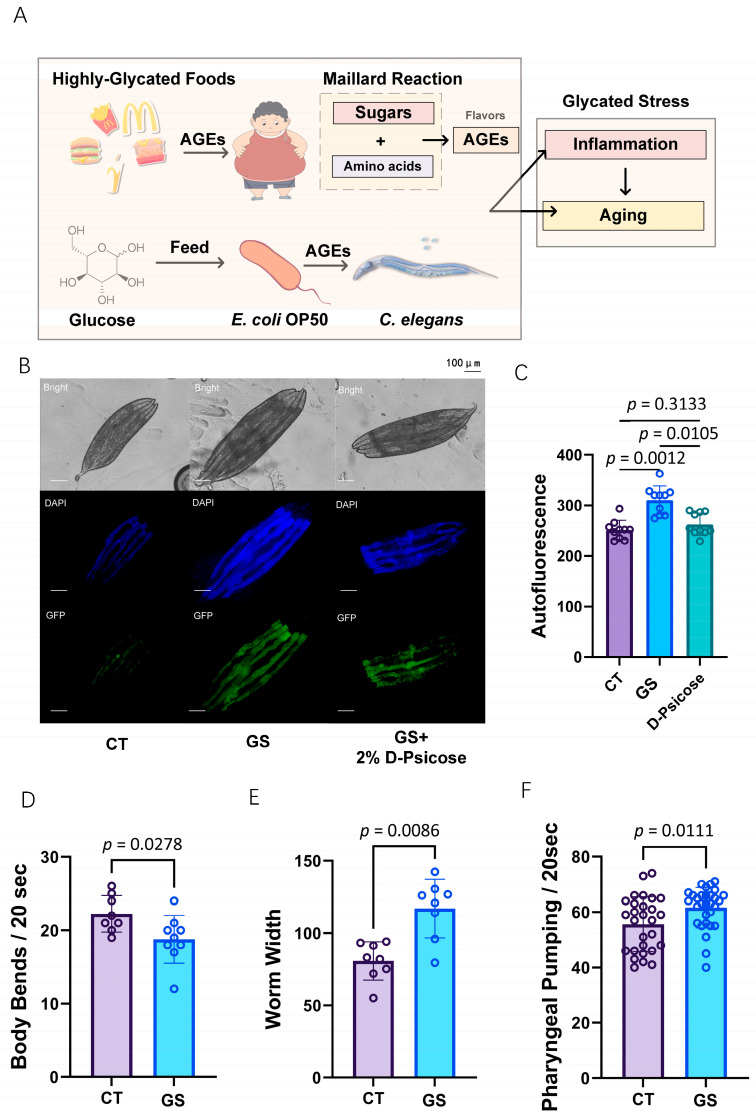
Evaluating the Impact of GS (GS) in *C. elegans*. (**A**) Schematic overview showing the formation and impacts of GS and AGEs. This panel depicts the dietary sources of AGEs, the Maillard reaction producing AGEs, and the subsequent GS leading to inflammation and aging in organisms. (**B**) Representative images of *C. elegans* under different treatments. The top row shows bright-field images, the middle row features DAPI staining of nuclei, and the bottom row illustrates GFP expression in worms. These images compare to control (CT), GS group, and GS plus 2% D-Psicose fed groups. The scale bar for panel B is 100 μm. (**C**) Quantification of autofluorescence levels across different groups, demonstrating the reduction of AGE accumulation when D-Psicose is included in the diet. The original data for this panel can be found in Supplementary Table S2. (**D**) Measurement of body bends per 20 s indicating the locomotor activity of worms under different treatments. The original data for this panel can be found in Supplementary Table S3. (**E**) Worm width, reflecting potential morphological changes induced by GS and the effect of D-Psicose. The original data for this panel can be found in Supplementary Table S4. (**F**): Pharyngeal pumping rates over 20 s, highlighting the effects of GS on feeding behavior in *C. elegans*. The original data for this panel can be found in Supplementary Table S5.

To simulate GS (GS) similar to that experienced in human glycation diets, we cultured Escherichia coli strain OP50 in a glucose-enriched medium to ensure complete glycation of its protein components (Figure 3A). This method replicates the formation of advanced glycation end-products (AGEs) that occur in human diets. The glycated OP50 was then fed to the worms, effectively inducing GS. 

In our exploration of the glycative stress model in *C. elegans*, we observed significant changes in both physiological and morphological characteristics as a result of diets in the GS group. Autofluorescence analysis, serving as a biomarker, revealed that worms with GS exhibited a notable increase in autofluorescence, signaling increased AGE accumulation compared to the control group (Figure 3B,C, *p* = 0.0012). This increase was mitigated by introducing 2% D-psicose to the GS diet, which significantly reduced autofluorescence levels (*p* = 0.0105), indicating the potential of D-psicose to inhibit AGE accumulation (Figure 3C). D-psicose, a positive compound in dietary restriction (CR), is known to reduce food intake and is a promising alternative in the management of GS, which can contribute to the observed effects. 

Additionally, the GS group showed a decrease in body bends (Figure 3D) and an increase in worm width (Figure 3E), indicating stress-induced morphological changes. These alterations in movement and body shape in the worms’ parallel observations in human populations consuming high-sugar diets, where reduced physical activity and increased body mass are common. The decrease in body bends suggests a decline in overall mobility, akin to the sedentary lifestyle often associated with excessive dietary sugar intake in humans. Similarly, the increase in worm width may reflect the tendency of increased body fat and weight gain seen in humans under similar dietary stresses.

Furthermore, pharyngeal pumping rates, a measure of feeding behavior, were increased in worms subjected to GS diets (Figure 3F, *p* = 0.0111). This increase in pharyngeal pumping suggests a potentially addictive response to the glycated diet, paralleling the addictive tendencies humans exhibit toward glycation-rich foods. The heightened pharyngeal activity indicates a possible craving or dependency mechanism in *C. elegans*, similar to human dietary patterns where high-sugar, highly glycated foods can lead to addictive consumption behaviors.

### 2.4. GS Induces Premature Aging in C. elegans

The application of the WormCNN model to classify and predict the biological age of *C. elegans* under different experimental conditions, specifically under control and GS environments, has provided significant insights into the effects of AGEs on worm aging. 

**Figure 4:**
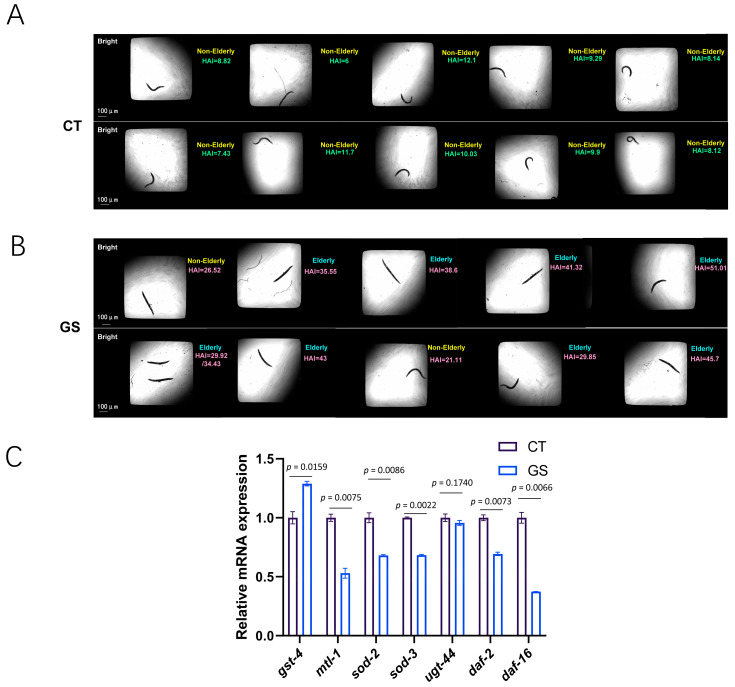
Performance of WormCNN in Classifying and Predicting the Biological Age of *C. elegans* under Control and GS Conditions. A/B: Panels (**A**,**B**) display representative images of *C. elegans* processed by the WormCNN, illustrating the classification of non-elderly and elderly worms, respectively, under control (CT) and GS (GS) conditions. Error bars represent the standard error of the mean (SEM), and experiments were performed in triplicate. Each image details the predicted biological age, showcasing the model’s accuracy in different experimental setups. (**C**) Presents the results of RT-qPCR analysis, showing the relative mRNA expression levels of various age-related genes (*gst-4, mtl-1, sod-2, sod-3, ugt-44, daf-2,* and *daf-16*) in control versus GS conditions. Statistical significance is indicated, highlighting the impact of GS on gene expression linked to aging. All primers used in the RT-qPCR experiments are listed in Supplementary Table S6. Statistical significance was determined using a two-tailed Student’s *t*-test for pairwise comparisons. *p*-values less than 0.05 were considered statistically significant.

As illustrated in Figure 4A,B, the model effectively differentiated between non-elderly and elderly worms, showing a clear distinction in the predicted biological ages between the control and GS groups. This disparity was more pronounced in the AGEs group, where worms exhibited substantially older age predictions, suggesting accelerated aging due to GS.

Further supporting these findings, RT-qPCR results (Figure 4C) confirmed the predictive outcomes of the WormCNN model. The analysis revealed marked changes in the expression of aging and stress-related genes such as *gst-4*, *mtl-1*, *sod-2*, *sod-3*, *ugt-44*, *daf-2*, and *daf-16*. Notably, the expression levels of *daf-2* and *daf-16*, key regulators of the insulin/IGF-1 signaling pathway, were significantly altered under GS. This reflects a stress-induced modulation of longevity pathways [29,30]. These findings were statistically significant, with *p*-values indicating a strong association between GS and gene expression alterations associated with aging.

## 3. Discussion

GS, resulting from the accumulation of AGEs, has been closely linked to the aging process [31,32,33]. AGEs are formed through non-enzymatic reactions between reducing sugars and proteins, leading to structural and functional alterations in biomolecules. This process is accelerated under high glucose conditions and has been implicated in various age-related diseases and metabolic disorders. The harmful effects of AGEs are well-documented, including the promotion of oxidative stress and inflammation, both of which contribute to the aging process and associated health decline.

In this study, we introduced an approach to inducing GS in *C. elegans* by culturing *E. coli* strain OP50 in a glucose-enriched medium. This method ensured the complete glycation of bacterial protein components, creating a more realistic model of dietary glycation that mimics human dietary patterns. Previous studies primarily used single protein and glucose cross-linking to produce AGEs, which does not fully capture the complexity of glycation in typical human diets.

To assess the impact of GS on worm aging, we utilized the WormCNN model to classify and predict the biological age of *C. elegans* under control and GS conditions. The WormCNN model effectively differentiated between non-elderly and elderly worms, with the GS group exhibiting substantially older age predictions. This indicates that GS significantly accelerates the aging process in *C. elegans.*

Furthermore, we introduced the Healthy Aging Index (HAI) as a new metric to quantify the biological health of worms. The HAI is calculated by comparing the predicted biological age of worms, as determined by WormCNN, against normative data derived from control conditions. This index provides a precise and automated measure of aging health, enabling a more comprehensive assessment of how GS affects aging in *C. elegans*. The results revealed that worms exposed to GS displayed significantly lower HAI scores, reinforcing the notion that GS has a detrimental effect on health and accelerates the aging process.

Building on these insights, our observations revealed that the intake of D-psicose could alleviate the accumulation of AGEs in *C. elegans*. As a beneficial sugar substitute in caloric restriction (CR) diets, this study confirms the potential of D-psicose to mitigate GS effects. Consequently, we recommend D-psicose as a viable dietary intervention to reduce GS-related damage, promote healthier aging profiles, and potentially extend lifespan.

It is essential to highlight some significant phenotypic changes observed. Worms under GS exhibited decreased mobility, characterized by reduced body bending, and an increase in body width. These physical changes are indicative of accelerated aging and stress. These morphological alterations pave the way for further intriguing findings regarding worm behavior.

Moreover, our experimental results revealed that the pharyngeal pumping rate, a measure of feeding behavior, was significantly increased in worms subjected to GS diets. This heightened pumping rate suggests a potential addictive response to the glycated diet, mirroring human tendencies towards addiction to high-sugar, highly glycated foods. In contrast, the control group displayed significantly slower intake rates. The increased pharyngeal pumping indicates that glycation diets lead to accelerated aging and decreased vitality in worms. The addictive nature of GS promotes excessive caloric intake, disrupting the balance between energy consumption and expenditure, which ultimately could lead to obesity.

In previous research, the concept of “Obesogenic potentials of artificial sweeteners” was proposed. Similarly, we hypothesize that the observed increase in pharyngeal pumping could be linked to the role of neurotransmitters such as serotonin (5-HT) and dopamine (DA) [34,35]. In worms, 5-HT acts on SER-7 receptors in motor neurons, activating the Gsα signaling pathway and promoting the release of acetylcholine (ACh) from MC neurons, thereby stimulating feeding behavior. Dopamine is known to play a fundamental role in the pleasure and addiction aspects of feeding behavior in mammals, potentially triggering increased food intake. We hypothesize that AGEs may upregulate *ser-7* and *gsα-1*, stimulating the release of 5-HT and ACh, which could explain the heightened pharyngeal activity observed. Furthermore, *egl-4*, which encodes a cyclic GMP-dependent protein kinase required for satiety-induced quiescence, might be downregulated by AGEs, reducing the sensation of fullness in worms. These hypotheses suggest that neuroregulation plays a critical role in the lipid metabolism and behavior of worms under GS, although further studies are required to confirm these mechanisms.

Interestingly, despite the observed premature aging, both *daf-2* and *daf-16* expression levels were reduced under GS. DAF-2 and DAF-16 are key components of the insulin/IGF-1 signaling pathway, which regulates longevity and stress responses in *C. elegans* [10,36,37].

The simultaneous reduction of both *daf-2* and *daf-16* indicates that glycation stress could be affecting the regulation of this pathway at multiple levels, possibly through feedback inhibition or by triggering alternative stress response mechanisms. In particular, the downregulation of *daf-16*, despite its known role in promoting longevity, might contribute to the accelerated aging phenotype we observed, highlighting the complexity of the organism’s response to glycation stress.

Given the complexity of this regulatory mechanism, further investigation is warranted to fully understand the interplay between GS and the insulin/IGF-1 signaling pathway. While this study provides initial insights into the effects of GS on aging, a more comprehensive exploration of these molecular mechanisms will be the focus of our future research.

Furthermore, the upregulation of genes like *gst-4* and *sod-2/sod-3*, which encode glutathione S-transferase and superoxide dismutase, respectively, points to a heightened cellular response to oxidative stress, potentially triggered by AGE accumulation. Similarly, the altered expression of *mtl-1* and *ugt-44* could reflect an adaptive response to detoxify and eliminate harmful glycation byproducts, enhancing the worms’ survival under glycation-induced stress conditions.

In conclusion, our study highlights the impact of GS on worm aging. The innovative method of using glycated OP50 bacteria offers a more accurate model of dietary glycation, enhancing the relevance of our findings to human health. The application of WormCNN demonstrates the power of integrating computational models with experimental biology to uncover insights into aging processes. While our findings provide valuable hypotheses regarding the neuroregulatory mechanisms underpinning glycation-induced aging, further research is necessary to fully elucidate these complex interactions. Additionally, our study recommends D-psicose as a dietary intervention to counteract the effects of GS.

## 4. Materials and Methods

### 4.1. Maintenance and Experimental Setup for C. elegans 

#### 4.1.1. Culturing Worms

Wild-type *C. elegans* strain N2, procured from the *Caenorhabditis* Genetics Center (Minneapolis, MN, USA), was propagated on a nematode growth medium (NGM). The NGM was prepared by combining 3 g of NaCl, 2.5 g of BactoPeptone, and 20 g of Agar in 1 L of double-distilled water (ddH_2_O). After autoclaving the NGM, it is cooled to 55 °C–60 °C (when the bottle can be comfortably handled), and at this point, 25 mL of PPB, 1 mL of 1M MgSO_4_, and 1 mL of 1M CaCl_2_ along with cholesterol solution (5 mg/mL) are added and thoroughly mixed.

The worms were synchronized using a standard bleach method. This synchronization involved a mixture of distilled water, 5M NaOH, and 10% sodium hypochlorite (NaClO), effectively isolating eggs from adult worms.

In addition to cultivation on solid media, the N2 strain can also be cultured in liquid media using S medium, which supports better dispersal and growth for certain experimental setups. The preparation of S Basal for 1 L of S medium includes dissolving 5.86 g of NaCl, either 1.31 g of K_2_HPO_4_·3H2O or 0.86 g of K_2_HPO_4_, and 6 g of KH_2_PO_4_. Post-dissolution. This mixture is subsequently autoclaved to ensure sterility. After cooling, additional components are introduced to complete the S medium formulation: 10 mL of 1M potassium citrate (pH adjusted to 6.0 with citric acid), 10 mL of trace metals solution, 3 mL of 1M CaCl_2_, and 3 mL of 1M MgSO_4_, which are essential for maintaining optimal ionic balance and worm health. All of the above reagents were purchased from Solarbio (Beijing, China).

Additionally, D-Psicose (Sigma, Shanghai, China), was prepared as a 2% solution and incorporated into the solid NGM to evaluate its effects on the GS worm.

**Figure 5:**
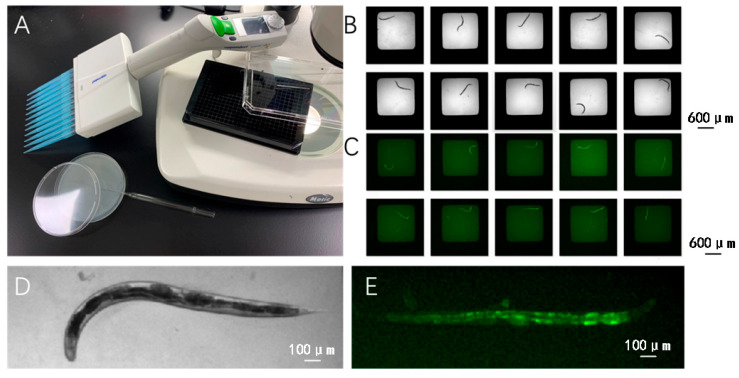
Experimental Setup and Imaging of *C. elegans*. (**A**) Overview of the laboratory setup showing the microscope, and the 384-well plate used for live imaging of *C. elegans*. (**B**) A sequence of images displaying various postures and developmental stages of *C. elegans* in a 384-well plate. (**C**) Fluorescent images demonstrating the autofluorescence of the intestinal tract in *C. elegans* within the liquid culture medium. (**D**) Close-up view of an individual *C. elegans* in liquid culture. (**E**) A detailed fluorescent microscopy image showing intense autofluorescence in the gut of *C. elegans*.

For experimental observations, worms are distributed into 384-well plates, with each well containing one or two worms. This setup facilitates high-throughput imaging and individual analysis, enabling detailed observation of phenotypic and behavioral traits under various experimental conditions. Figure 5A–E illustrate the setup and examples of worm placement within the wells, providing visual support for the described methodology.

#### 4.1.2. Glycation Induction in Bacterial Cultures

Initially, a 50 mL centrifuge tube was prepared by adding 0.6 g of glucose to 30 mL of Luria-Bertani Broth (LB broth), ensuring the sugar was fully dissolved to create a glucose-enriched environment. A single colony of *Escherichia coli* OP50, widely used in *C. elegans* research due to its well-characterized interactions with the worm, was isolated from an LB agar plate and inoculated into the prepared medium.

This glucose-supplemented LB medium was then incubated in an orbital shaker at 37 °C for 16 h. This duration was chosen to promote bacterial growth while allowing sufficient time for glycation processes to occur. Post-incubation, the bacteria were harvested for biochemical analysis to assess AGE formation and other glycation-related changes.

#### 4.1.3. Detection of Advanced Glycation End-Products (AGEs)

Advanced Glycation End-Products (AGEs), known for their characteristic autofluorescence, were detected using fluorescence spectroscopy (ZEISS Axioscope 5, Shanghai, China). AGEs such as pentosidine, crosine, and pyropyidine exhibit specific fluorescence properties, with excitation and emission wavelengths ranging from 335/385 nm to 379/463 nm, respectively [38]. 

To facilitate the normalization of sample concentrations, 1 mL of both glycated and control OP50 bacterial samples were collected in 1.5 mL Eppendorf tubes (Shanghai, China), and their optical density was measured at 600 nm (OD600). 

For the fluorescence measurements, 300 μL of each sample was aliquoted into duplicate wells of a 384-well plate. A plate reader, set to the appropriate wavelength combinations for detecting the AGEs of interest, measured the fluorescence intensities. Typically, samples from glycated cultures displayed significantly higher fluorescence intensities, indicative of successful AGE formation.

#### 4.1.4. GS Model in *C. elegans*

Following the bacterial AGE analysis, the methodology was adapted to study the effects of GS on *C. elegans*. Cultured OP50 bacteria, enriched in AGEs, were concentrated by centrifugation to a final volume of 10 mL. 

These bacteria were then used to feed worms at the young adult (YA) stages. A 2% agarose solution was heated until fully dissolved, and one or two drops were placed on a microscope slide, followed by the placement of a cover slip. 

After the agarose had solidified, 1 µL of 16 mM levamisole hydrochloride (Sigma, Shanghai, China) was added. Subsequently, five to six worms were transferred onto the slide. Once the worms were immobilized, they were arranged neatly for observation under a fluorescence microscope equipped with a DAPI (ZEISS Axioscope 5, Shanghai, China) filter. This setup allowed for the detection of pronounced blue autofluorescence in the worms’ guts, confirming the uptake and metabolic assimilation of the glycated compounds. 

#### 4.1.5. RT-qPCR Analysis

For quantitative real-time PCR (RT-qPCR) analysis, total RNA was extracted from worms using the TRIzol reagent (Tsingke, Beijing, China) according to the manufacturer’s instructions. The integrity and concentration of RNA were assessed using a NanoDropONE (Thermo Fisher Scientific, MA, USA). Complementary DNA (cDNA) was synthesized from 1 µg of total RNA using a reverse transcription kit (Tsingke, Beijing, China) following the protocol provided.

RT-qPCR was performed using a SYBR Green PCR Master Mix (Tsingke, Beijing, China) on a real-time PCR system (QuantStudio 3, Thermo Fisher Scientific, Waltham, MA, USA). The thermal cycling conditions were as follows: initial denaturation at 95 °C for 10 min, followed by 40 cycles of 95 °C for 15 s, and 60 °C for 1 min. Each sample was run in triplicate to ensure the accuracy and reproducibility of the results.

Gene-specific primers were used to amplify target genes related to aging and stress response pathways. The expression levels of the genes were normalized to the housekeeping gene actin. Relative gene expression was calculated using the 2^−ΔΔCT^ method, which provides a quantitative measure of the relative abundance of target mRNA in the samples.

All primers used in the RT-qPCR experiments are listed in Supplementary Table S6 and are available for reference. This table provides detailed sequences and annealing temperatures for each primer pair, ensuring the transparency and reproducibility of the experimental conditions.

### 4.2. Worm Imaging Collection and Analysis

#### 4.2.1. Worm Image Collection

To monitor the lifespan and assess the biological age and aging status of *C. elegans* housed in a 384-well plate setup, worms were individually cultured in each well, with each well serving as a separate sample and labeled accordingly. Daily imaging sessions were conducted at consistent times to track these isolated subjects. Dead worms were systematically excluded from the dataset to ensure that the deep learning model used for predicting biological lifespan was trained exclusively on viable subjects. 

For model prediction tasks related to lifespan and aging assessments using deep learning models, worms from both solid and liquid media cultures were meticulously transferred into the designated wells of the 384-well plates on day 5, specifically for these imaging sessions. The strict requirement of one worm per well is not necessary. This careful handling ensured accurate and consistent data collection, which is vital for the reliability of our aging assessments.

Images were captured using fluorescence microscope equipped with a 10× objective. The protocol involved capturing brightfield channel scans of each well, optimizing the exposure time to 30 milliseconds per frame. The camera was configured in high-resolution mode, achieving a resolution of 2048 × 2048 pixels to preserve detailed image quality. A standard halogen lamp provided consistent illumination, minimizing phototoxic effects. Image acquisition was regulated and synchronized using Zeiss Zen (version 2.3) software, with each image timestamped for precise chronological alignment with experimental data.

#### 4.2.2. Segmentation of Worms

The segmentation of the central line in individual worms was performed using a series of image-processing techniques as below. Each worm was isolated in a 384-well plate and photographed to ensure clear visibility and consistency across samples. The images were initially annotated using the LabelMe library (5.4.1) with Python (3.7) to define the regions of interest.

Subsequent image processing was carried out using the OpenCV library (4.9.0) in Python. This process included median filter denoising to remove image noise without blurring important edges, contrast enhancement through histogram equalization to improve image visibility, and conversion of images to 8-bit grayscale to simplify the data for analysis.

**Figure 6:**
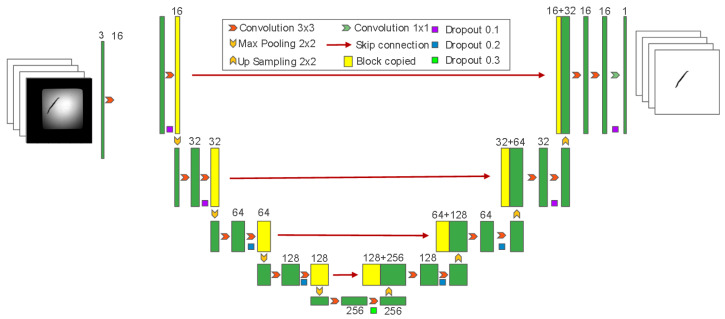
U-Net architecture for worm segmentation. The network features a contracting path for feature extraction through convolutional layers and downsampling, a bottleneck for high-level feature representation, and an expansive path for upsampling and precise localization, ultimately producing segmented images highlighting the worm’s central line.

To accurately segment the central line of the worms, we employed a deep learning-based approach utilizing a U-Net architecture (Figure 6). U-Net is well-known for its effectiveness in biomedical image segmentation, owing to its ability to accurately describe intricate structures in noisy biomedical images.

For training and optimizing the U-Net model (Figure 2), we assembled a dataset of annotated worm images from both solid and liquid culture media. These images were augmented using various data augmentation techniques to enhance the model’s robustness and ability to generalize across different conditions. The deep learning model was developed and refined using PyTorch (2.2.0), with hyperparameter tuning conducted through cross-validation and the Hyperopt package (0.2.7). This systematic approach ensures precise segmentation and contributes significantly to the subsequent analysis of worm morphology and physiology.

#### 4.2.3. Worms Image Extraction and Reconstruction

In this study, we employed advanced computer vision techniques to process and analyze images of *C. elegans*, focusing on extracting and reconstructing the worm’s central line. Initially, the grayscale image of the *C. elegans* was read using OpenCV. We then applied a binary threshold to isolate the worm from the background. This was achieved using the cv2.threshold function, where pixels with intensity values below a threshold were set to the maximum value, and all other pixels were set to zero:

(1) $$\left\{\begin{array}{c} 255\;\;if\;I\left(x,y\right)<128\\ 0\;\;otherwise \end{array}\right.$$

To refine the binary image and remove small artifacts, we performed morphological closing followed by opening using a 3 × 3 kernel. This step helps in bridging small gaps and removing noise:

(2) $$Closing=dilate\left(erode\left(thresh\right)\right)$$

(3) $$Opening=erode\left(dilate\left(closing\right)\right)$$

The binary image was skeletonized using the skeletonize function from the *skimage.morphology* module. This process reduces the binary image to a single-pixel-wide representation of the worm’s central line:

(4) $$skeleton=skeletonize\left(opening/255\right)$$

From the skeletonized image, we extracted the coordinates of the skeleton pixels. Key points were selected at regular intervals to represent the worm’s central line more sparsely. We used linear regression to determine the global direction of the worm. By fitting a line to the key points, we obtained the slope and intercept, from which the global orientation angle was calculated:

(5) y=mx+b

(6) $$globalangle=arctan\left(m\right)$$

We developed a function to segment and reconstruct the worm image based on the skeleton coordinates and the global direction. Each segment was extracted by rotating the coordinate frame to align with the global orientation:

(7) $$x^{\prime }=xcos\left(\theta \right)-ysin\left(\theta \right)$$

(8) $$y^{\prime }=xsin\left(\theta \right)+ycos\left(\theta \right)$$

The processed images, including the original, thresholded, skeletonized, and reconstructed worm images, were visualized using Matplotlib. 

### 4.3. WormCNN Architecture

WormCNN is a deep learning model based on a series of convolutional layers, batch normalization, activation functions, and a global average pooling layer that culminates in a fully connected layer for binary classification. 

To ensure transparency and facilitate reproducibility of our findings, the code used in the study, including scripts for data preprocessing and the WormCNN model implementation, can be accessed at https://github.com/Marissapy/WormCNN (accessed on 3 September 2024). This repository provides comprehensive documentation and source code, enabling other researchers to replicate our results and further explore the methodologies employed in our analysis.

#### 4.3.1. Convolutional Layers

The initial layer of WormCNN utilizes a 3 × 3 kernel to convolve 32 filters with the input image, which is padded to maintain the dimensionality through the layers. This is followed by batch normalization and a ReLU activation function:

(9) $$out=ReLU\left(BatchNorm\left(Conv\left(x\right)\right)\right)$$

The ReLU (Rectified Linear Unit) function is applied to the output of the first dense layer. ReLU is defined as:

(10) $$ReLU\left(x\right)=max\left(0,x\right)$$

#### 4.3.2. Residual Block

The network incorporates a custom residual block designed to deepen the feature extraction without losing significant information from the input through the layers. This block contains two convolutional layers, with each followed by batch normalization and ReLU activation. The output of the residual block is added to the input of the block to form the final output of the block, facilitating the learning of identity functions and mitigating the vanishing gradient problem.

(11) $$resout=ReLU\left(BatchNorm\left(Conv\left(ReLU\left(BatchNorm\left(Conv\left(x\right)\right)\right)\right)+x\right)\right)$$

#### 4.3.3. Global Average Pooling

Following the convolutional and residual layers, a global average pooling layer is applied to reduce each feature map to a single average value, reducing the dimensionality and preparing the feature representation for classification.

(12) $$gap=GlobalAvgPool\left(resout\right)$$

#### 4.3.4. Fully Connected Layer

The output from the global average pooling is flattened and passed through a fully connected layer with 64 neurons, followed by a ReLU activation. This is connected to a final sigmoid-activated layer that outputs the probability of the worm being classified as old or young.

(13) $$fc_{out}=Sigmoid\left(Dense\left(ReLU\left(Dense\left(gap\right)\right)\right)\right)$$

The mathematical expression for the sigmoid function is:

(14) $$\sigma \left(x\right)=\frac{1}{1+e^{-x}}$$

To extend the utility of the described model, it is also applicable to lifespan regression models in *C. elegans*. By employing the worms cultured in liquid media as a baseline for “health”, the lifespan conditions under these settings serve as the standard for assessing “healthy” aging. The model uses the fully connected layer outputs to regress the biological lifespan, quantifying it as the “Healthy Aging Index” (HAI). This index provides a quantifiable measure of the worm’s aging health, comparing each individual’s predicted biological age against the normative data derived from the liquid culture lifespan metrics.

### 4.4. Training Procedure

The WormCNN was trained using a custom dataset of worm images labeled according to the age of the worms. The images were preprocessed through a series of transformations, including resizing to a fixed dimension (600 × 30 pixels), conversion to grayscale, and normalization.

The training procedure involved minimizing the binary cross-entropy loss between the predicted labels and the true labels of the images using the Adam optimizer. The training was conducted over 64 epochs, with the performance metrics including loss and accuracy, monitored for both training and validation datasets.

### 4.5. Model Validation

The validation of the models was conducted using key metrics to assess classification performance: accuracy, precision, recall, and the F1-score. These metrics are crucial for evaluating the accuracy and effectiveness of the model in identifying and classifying data correctly.

(15) $$Accuracy=\frac{TP+TN}{TP+FP+TN+FN}$$

(16) $$Precision=\frac{TP}{TP+FP}$$

(17) $$Recall=\frac{TP}{TP+FN}$$

(18) $$F1=2*\frac{Precesion*Recall}{Precision+Recall}$$

where TP represents the count of true positives, TN denotes the count of true negatives, FP is the number of false positives, and FN stands for the number of false negatives.

### 4.6. Statistical Analysis

All statistical analyses were performed using GraphPad Prism (10.1.0). Survival curves were generated using the Kaplan-Meier method, and statistical significance was assessed using the log-rank (Mantel–Cox) test. 

For comparisons between multiple groups, one-way ANOVA followed by Tukey’s post hoc test was applied. If the data did not meet the assumptions for ANOVA, a non-parametric Kruskal–Wallis test followed by Dunn’s multiple comparisons test was employed. Statistical significance was determined at a *p*-value of < 0.05. All data are presented as mean ± standard error of the mean (SEM), and each experiment was conducted in triplicate to ensure reproducibility.

## 5. Conclusions

This study offers a analysis of the effects of GS on aging in *C. elegans*, using a model induced by dietary advanced glycation end-products (AGEs). Our analysis with WormCNN revealed that GS speeds up the aging process, as indicated by increased autofluorescence, changes in aging-related gene expression, and shifts in behavior. D-psicose, when introduced as a dietary intervention, demonstrated potential in reducing AGE accumulation, suggesting a promising avenue for future research on dietary strategies to combat glycation-induced aging. 

The combination of WormCNN’s deep learning with traditional assays was crucial in quantifying aging markers and establishing a Healthy Aging Index (HAI), highlighting the importance of computer vision in aging studies. This research enhances our understanding of aging processes, and paves the way for interventions aimed at promoting healthy aging and preventing age-related diseases. Future studies will explore the molecular mechanisms of GS on aging and investigate additional dietary interventions to counteract its adverse effects.

## Figures and Tables

**Table 1:** The U-Net model’s performance metrics for segmenting worms.

Metric	Value
True Positive (TP)	35,610
False Positive (FP)	7527
True Negative (TN)	12,932,412
False Negative (FN)	579
Sensitivity (TPR)	98.40%
Specificity (TNR)	99.94%
Overall Accuracy	99.94%
Precision (PPV)	82.55%
F1-Score	89.78%

**Table 2:** Performance metrics from the WormCNN model for classifying *C. elegans* based on a 20% survival threshold.

Metric	Value
True Positives (TP)	54,100
False Positives (FP)	1509
True Negatives (TN)	547,099
False Negatives (FN)	10,640
True Positive Rate (TPR)	83.6%
Precision (PPV)	97.3%
Specificity (TNR)	99.72%
Overall Accuracy	98.0%
F1-Score	89.91%

## Data Availability

All code and data used in this study can be accessed at https://github.com/Marissapy/WormCNN (accessed on 3 September 2024).

## Supplementary Materials

The following supporting information can be downloaded at: https://www.mdpi.com/article/10.3390/ijms25179675/s1.
